# The application of a network approach to Health-Related Quality of Life (HRQoL): introducing a new method for assessing HRQoL in healthy adults and cancer patients

**DOI:** 10.1007/s11136-015-1127-z

**Published:** 2015-09-14

**Authors:** Jolanda J. Kossakowski, Sacha Epskamp, Jacobien M. Kieffer, Claudia D. van Borkulo, Mijke Rhemtulla, Denny Borsboom

**Affiliations:** Department of Psychology, University of Amsterdam, Weesperplein 4, 1018 WZ Amsterdam, The Netherlands; Division of Psychosocial Research and Epidemiology, The Netherlands Cancer Institute, Plesmanlaan 121, 1066 CX Amsterdam, The Netherlands; Interdisciplinary Center Psychopathology and Emotion Regulation, University Medical Center Groningen, University of Groningen, Hanzeplein 1, 9700 RB Groningen, The Netherlands

**Keywords:** Health-Related Quality of Life, Cancer, Network analysis, Psychometrics, Short Form Health Survey, SF-36

## Abstract

**Purpose:**

Health-Related Quality of Life (HRQoL) research has typically adopted either a formative approach, in which HRQoL is the common *effect* of its observables, or a reflective approach—defining HRQoL as a *latent variable* that determines observable characteristics of HRQoL. Both approaches, however, do not take into account the complex organization of these characteristics. The objective of this study was to introduce a new approach for analyzing HRQoL data, namely a network model (NM). An NM, as opposed to traditional research strategies, accounts for interactions among observables and offers a complementary analytic approach.

**Methods:**

We applied the NM to samples of Dutch cancer patients (*N* = 485) and Dutch healthy adults (*N* = 1742) who completed the 36-item Short Form Health Survey (SF-36). Networks were constructed for both samples separately and for a combined sample with diagnostic status added as an extra variable. We assessed the network structures and compared the structures of the two separate samples on the item and domain levels. The relative importance of individual items in the network structures was determined using centrality analyses.

**Results:**

We found that the global structure of the SF-36 is dominant in all networks, supporting the validity of questionnaire’s subscales. Furthermore, results suggest that the network structure of both samples was highly similar. Centrality analyses revealed that maintaining a daily routine despite one’s physical health predicts HRQoL levels best.

**Conclusions:**

We concluded that the NM provides a fruitful alternative to classical approaches used in the psychometric analysis of HRQoL data.

## Introduction

The question of how theoretical constructs like Health-Related Quality of Life (HRQoL) should be related to observables reflects one of the fundamental scientific issues facing any field: how should we think about the relation between constructs and observables? Two dominant approaches to this question are known as formative and reflective modeling [1, 2]. In formative models (FMs), items are viewed as causes of the theoretical construct under consideration, whereas in reflective measurement models (RMMs), items are seen as effects of that construct. In the present paper, we argue that neither of these approaches suits HRQoL, and present an alternative approach based on a network model (NM).

**Fig. 1:**
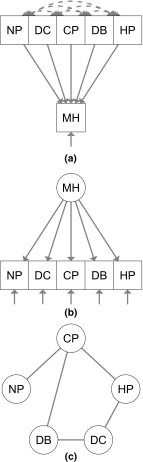
Examples of an FM (**a**), RMM (**b**) and an NM (**c**) that can be applied to HRQoL. *FM* formative model; *RMM* reflective measurement model; *NM* network model; *MH* mental health; *NP* item 9b of the 36-item Short Form Health Survey (SF-36): “how much of the time during the past 4 weeks have you been a very nervous person”; *DC* item 9c of the SF-36: “how much of the time during the past 4 weeks have you felt so down in the dumps that nothing could cheer you up”; *CP* item 9d of the SF-36: “how much of the time during the past 4 weeks have you felt calm and peaceful”; *DB* item 9f of the SF-36: “how much of the time during the past 4 weeks have you felt downhearted and blue”; *HP* item 9h of the SF-36: “how much of the time during the past 4 weeks have you been a happy person”. Presumed causal relations between variables are displayed by *arrows*. Labels on covariances among observed variables and on variances between latent and observed variables are omitted for clarity of presentation

Some of the analyses performed in HRQoL research have been based on the application of FMs using principal components analysis (PCA), creating weighted composites of observables to achieve data reduction [3]. The 36-item Short Form Health Survey (SF-36), a commonly used instrument across different disease conditions and patient groups [4], has been developed on the basis of PCA. In an FM, HRQoL is the common *effect* of items (or simply a composite score formed out of them, like in PCA [5]). The idea behind the FM is that observed variables contribute to HRQoL: if the observables change, HRQoL changes as a result. A simplified example of the FM is represented in Fig. 1a where the observables are represented as forming a “mental health” (MH) component, one of the domains of the SF-36.

An advantage of the FM is that it allows people with similar levels of HRQoL to have different item responses. For example, John may have a poor HRQoL because he is a very nervous person, whereas Jane may have a poor HRQoL because she feels downhearted and blue. Furthermore, the FM is appropriate when one would like to calculate a single score to represent someone’s HRQoL, which can be used as an index of general functioning. However, the FM also has some downsides. First, the FM is unidentified unless external outcome variables are added to identify its parameters [6]. Since different external variables yield different modeling solutions, the definition of a formative construct cannot be assumed stable across applications (i.e., *interpretational confounding*; [7]). Second, since the FM does not have implications for the correlation structure between item responses, it cannot evaluate important relations between items that make up HRQoL, nor the processes that give rise to the correlation structure that characterizes it [1]: relations between items are modeled as nuisance, even when they may harbor important information.

An alternative to the FM [e.g., 8, 9] is the RMM. In an RMM, HRQoL is defined as the common determinant of item responses. For example, Fig. 1b shows that the items NP, DC, CP, DP and HP have a common determinant, namely MH. When using an RMM, one has to satisfy the assumption of *local independence* [e.g., 10, 11], which states that two variables are locally independent when controlling for a third (latent) variable. So, an RMM implies that a high correlation between responses like “feeling calm and peaceful” and “being a very nervous person” can be explained by the common influence of the latent variable MH. For this reason, the RMM is also called a *common cause model* [6].

However, it is questionable whether HRQoL should be represented this way. It seems conceptually implausible that having a poor HRQoL results in being downhearted and blue. The reverse has more potential [12, 13]: i.e., downhearted and blue contributes to having a poorer HRQoL. Furthermore, the assumption of local independence may be unrealistic. The correlation between CP and NP is probably not due to the central influence of MH, but more likely results from a direct connection between the two. Signifying that, although the RMM represents an important model for relations between observables, a feature not found in the FM, it is unlikely to be fully appropriate for HRQoL.

Thus, the FM is a model useful for constructing a general health index, but one that ignores the structure present in item connections. Contrary to the FM, the RMM does model relations between observables, but does so on unrealistic assumptions. Thus, both the FM and the RMM are not able to capture the complexity of the relationship between HRQoL and its observables. In other words, we currently have no satisfactory way of thinking about the relation between HRQoL and its observables. In this study, we argue that the novel perspective offered by the NM can fill this gap.

The NM has been introduced as a psychometric approach that offers an alternative to the RMM and the FM. In an NM, connections between observables are assumed to result from a system in which variables have direct (pairwise) interactions [14]. These interactions can reflect the influence of observables on each other via bidirectional, and potentially causal, relations: i.e., feeling downhearted and blue leads to that person feeling less calm and peaceful, which in turn can lead to that person feeling more downhearted and blue. Alternatively, these interactions may arise because variables are part of the same homeostatic system, or because described relations are conditional. In these cases, variables will become coupled: they show dependencies that will not vanish after conditioning on all other variables. The structure of these relations can be represented and analyzed using an NM.

Figure 1c shows a simplified example of an NM applied to HRQoL, in which DB is connected to CP, which, in turn, is connected to NP. Typically, the absence of a direct relation means that variables will become statistically independent when conditioning on the variables mediating the path between [15]. In Fig. 1c, NP and DP are independent after conditioning on CP. Importantly, within the NM, HRQoL is neither assumed to be a common effect (as in the FM) nor the common cause of item responses (as in the RMM): the NM offers a third alternative for conceptualizing construct–observation relations. This framework has already been fruitfully applied to intelligence [16], psychopathology [17] and personality research [18].

Importantly, instead of a causal relation, an NM assumes that the relation between individual item responses and the construct HRQoL is *mereological*; individual components are *part* of the construct, because the construct is understood as a network of mutually interacting variables that together form HRQoL [19]. As such, direct connections between item responses are not only accommodated in an NM, but form the flesh and bones of the network structure. Not only connections between item responses, but connections between health domains can be examined as well, as the SF-36 consists of eight domains, which form a profile of a person’s health status [4]. In this study, we aim to demonstrate that the NM can be successfully applied to HRQoL research and show that it provides a fruitful alternative to an RMM or FM: the NM provides novel ways of representing and analyzing connections present among items or domains, which suggest new avenues for research and may inform treatment interventions.

Next to providing a novel approach for operationalizing HRQoL, the NM allows researchers to ask new questions about item structures in relation to the construct. In this study, we investigate four such questions. First, we examine how HRQoL is structured in terms of its network architecture, by constructing networks of two Dutch samples of healthy and non-healthy individuals. Second, we exploratively examine the structure of HRQoL on domain level by constructing domain networks for each sample. Third, we investigate which items are most central to HRQoL by using network metrics of centrality. Fourth, we test whether the network structure of healthy and cancer populations are significantly different.

## Method

### Data source

This study involves a secondary analysis of data that were originally gathered for the International Quality of Life Assessment Project (IQOLA) and have been described in detail in a previous paper [20]. The data involved were unidentifiable; it could not be traced back to the individual. Therefore, we did not require informed consent. The present study focuses on two subsamples of the IQOLA project: Dutch cancer patients (cancer patient sample) and a Dutch nationwide sample of adults who were not diagnosed with cancer (national sample). Participants completed the SF-36 between 1992 and 1994 (cancer patient sample) or in 1996 (national sample). In addition, we combined the two datasets (combined sample) to analyze the function of diagnostic status (i.e., the distinction between being diagnosed with cancer or not) in the HRQoL system. To this end, we added diagnostic status as a separate variable in the network structure.

### Sf-36

The SF-36 [21, 22] is a HRQoL questionnaire that has been adapted and translated into more than ten languages over the past few decades, and has also been validated in various patient groups and languages [23–25]. The SF-36 is based on an FM and comprises the following eight first-order latent variables (domains): physical functioning (PF), role limitations–physical (RP), bodily pain (BP), general health (GH), vitality (VT), social functioning (SF), role limitations–emotional (RE) and mental health (MH). These domains are themselves modeled as the cause of two second-order latent variables [26], which are represented by physical and mental component summary scores (PCS and MCS, respectively). Domain scores were calculated by summing up item responses, after which the scores were transformed to range between 0 and 100. PCS and MCS scores were calculated using standard US scoring algorithms [27]. Item allocation to the eight domains identified by the SF-36 can be found in Table 1.

**Table 1:** Allocation of the 36-item Short Form Health Survey items to the eight domains of Health-Related Quality of Life

Domain	Items
Physical functioning	3a–3j
Role limitations–physical	4a–4d
Bodily pain	7 and 8
General health	1 and 11a–11d
Vitality	9a, 9e, 9g, 9i
Social functioning	6 and 10
Role limitations–emotional	5a–5c
Mental health	9b, 9c, 9d, 9f, 9h
Physical component summary	PF, RP, BP, GH
Mental component summary	VT, SF, RE, MH

Item 2 and diagnostic status are not included in a domain and are thus not shown

### Network analysis

An NM conceptualizes HRQoL as a network of mutually interacting characteristics [28]. NMs consist of two elements: nodes (circles; observed variables) and edges (lines; relations between variables [29]). To obtain a network, we estimated a Gaussian Graphical Model (GGM) [30] for all samples on both the item level and the domain level, a network in which an edge indicates a nonzero partial correlation between two nodes, while controlling for all other nodes in the network. This means that two connected nodes display a level of covariation that cannot be explained by other nodes in the network. To control for spurious connections that may arise due to multiple testing, and for the computational size of the problem, we applied the graphical lasso [31]: a form of lasso regularization [32], which utilizes penalized maximum likelihood estimation. The result is a sparse GGM in which many edge weights are set to zero and thus removed from the network. The network that is formed with a graphical lasso is therefore both interpretable and guarded against overfitting. The graphical lasso uses a tuning parameter to control the sparsity of the network, which we chose by minimizing the Extended Bayesian Information Criterion [EBIC; 33]. This methodology is explained in more detail by Costantini et al. [34]. Because the GGM assumes that the input covariance matrix comes from a population that follows a multivariate Gaussian density, whereas the SF-36 only measures at an ordinal scale, we computed the polychoric correlation matrix to apply the graphical lasso.

### Network comparison

We checked for differences in network structures by means of a permutation test developed by van Borkulo et al. [35]. The difference is defined as the deviation in absolute weighted sum scores of the connections [36]. This permutation-based test randomly regrouped participants from the cancer patient sample and the national sample repeatedly (1000 times) and calculated the differences between these subsamples. The resulting distribution under the null hypothesis (both subsamples are equal) is used to test the observed difference of the original subsamples against a significance level of 0.05. Both weighted network structures (taking the edges’ weights into account) and unweighted network structures (only taking the presence of an edge into account) were tested. The latter is tested to investigate whether the basic structure of the samples are similar, the first to investigate whether the strength of individual connections in the networks structures are similar.

### Centrality analysis

To analyze the place and function of items within individual networks, we use the measure of *closeness centrality* [37]. Edges between nodes are interpreted as paths: the stronger the edge, the stronger the path between relevant nodes, and the easier it is to travel from one node to another [34]. A highly central node is one from which it is possible to easily travel to all other nodes. Such paths may be interpreted as etiological progressions by which individual problems can lead to closely connected problems. In particular, nodes with high closeness centrality have a high ability to predict other nodes, and as such they may correspond to characteristics that have a particularly important function in HRQoL. Adopting the formal interpretation of HRQoL, when an item has a high closeness, it predicts HRQoL well.

All analyses were performed using the R statistical software 3.1.2. GGMs were constructed with the R-package huge version 1.2.6 [38]. Network visualization and the computation of centrality measures were done with the R-package qgraph version 1.3.1 [39].

## Results

### Participant characteristics

A total of 2227 participants completed the SF-36. As shown in Table 2, the mean age of the cancer patient sample (*N* = 485) was 57.27 years with 58 % women. The national sample (*N* = 1742) had a mean age of 46.71 years with 44 % women. Table 2 also shows the mean scores on the eight domains of the SF-36 for the individual samples.

**Table 2:** Means (SD) of the cancer patient sample and the national sample

Cancer patient sample				National sample			
Domain	Men	Women	Total	Domain	Men	Women	Total
PF	85.82 (20.30)	81.31 (23.67)	83.90 (21.87)	PF	67.73 (26.48)	64.12 (27.86)	65.58 (27.94)
RP	78.94 (33.87)	74.73 (38.12)	76.93 (35.84)	RP	53.13 (46.93)	42.80 (40.41)	46.97 (43.59)
BP	77.50 (22.52)	72.58 (23.53)	75.37 (23.12)	BP	72.78 (29.35)	67.20 (26.69)	69.46 (27.64)
GH	71.98 (20.24)	70.45 (20.43)	71.39 (20.30)	GH	51.08 (22.97)	50.97 (24.24)	51.01 (23.36)
VT	72.21 (17.94)	64.71 (19.61)	68.89 (19.01)	VT	63.75 (22.25)	53.56 (24.85)	57.68 (24.95)
SF	86.35 (20.86)	82.22 (23.53)	84.48 (22.09)	SF	77.50 (23.25)	71.61 (27.54)	73.99 (25.93)
RE	85.72 (29.77)	78.51 (35.82)	82.44 (32.86)	RE	74.17 (40.46)	60.45 (43.54)	65.99 (42.21)
MH	79.57 (16.20)	73.88 (18.29)	77.04 (17.35)	MH	76.90 (18.29)	69.36 (21.61)	72.40 (20.67)
PCS	37.03 (6.39)	36.23 (6.25)	36.63 (6.31)	PCS	50.53 (9.55)	49.31 (10.57)	50.00 (10.01)
MCS	42.84 (5.75)	43.21 (6.20)	43.07 (5.97)	MCS	51.28 (9.16)	48.36 (10.81)	50.00 (10.00)

*BP* bodily pain, *GH* general health, *MH* mental health, *PF* physical functioning, *RE* role limitations–emotional, *RP* role limitations–physical, *SF* social functioning, *VT* vitality, *PCS* physical component summary, *MCS* mental component summary

### Network analysis

**Fig. 2:**
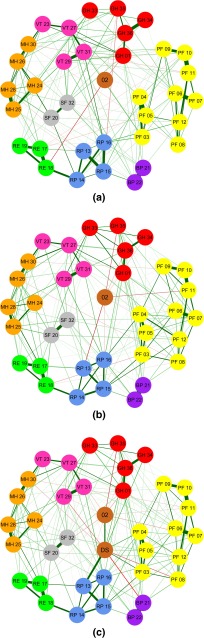
Network of Health-Related Quality of Life (HRQoL) as measured by the 36-item Short Form Health Survey in a cancer patient sample (**a**), a national sample (**b**) a pooled sample of the former two (**c**). The size of the absolute polychoric partial correlation between two nodes is represented using the color and thickness of an edge [37]. *Node colors* correspond to the eight domains: *RED* general health (GH), *YELLOW* physical functioning (PF), *ORANGE* mental health (MH), *BLUE* role limitations–physical (RP), *GREEN* role limitations–emotional (RE), *PURPLE* bodily pain (BP), *GREY* social functioning (SF), *PINK* vitality (VT), *BROWN* items not belonging to a domain

Figure 2a–c show networks of the cancer patient sample, the national sample and the combined sample, respectively. Edges between nodes within a network correspond to polychoric partial correlations between items, controlling for all other items. The stronger a connection between two nodes, the thicker and more saturated the edge. Positive and negative connections are denoted by green and red edges, respectively [34]. Each node corresponds to a single SF-36 item (as given in Table 3) and is colored according to the domain it is allocated to (as given in Table 1). Item 2 and the diagnostic status variable are not part of any domain and thus are represented as separate. The Fruchterman–Reingold algorithm, which places more strongly connected nodes closer together, is used for node placement in all networks [40].

**Table 3:** Items of the 36-item Short Form Health Survey and their assigned labels

Item	Item label	Domain color	Item meaning
1	GH 01	Red	In general, how would you say your health is?
2	02	Brown	Compared to 1 year ago, how would you rate your health in general now?
3a	PF 03	Yellow	Does your health limit you in vigorous activities, such as running, lifting heavy objects, participating in strenuous sports?
3b	PF 04	Yellow	Does your health limit you in moderate activities, such as moving a table, pushing a vacuum cleaner, swimming or cycling?
3c	PF 05	Yellow	Does your health limit you in lifting or carrying groceries?
3d	PF 06	Yellow	Does your health limit you in climbing several flights of stairs?
3e	PF 07	Yellow	Does your health limit you in climbing one flight of stairs?
3f	PF 08	Yellow	Does your health limit you in bending, kneeling or stooping?
3g	PF 09	Yellow	Does your health limit you in walking more than one kilometer?
3h	PF 10	Yellow	Does your health limit you in walking a few hundred meters?
3i	PF 11	Yellow	Does your health limit you in walking one hundred meters?
3j	PF 12	Yellow	Does your health limit you in bathing or dressing yourself?
4a	RP 13	Blue	Did you cut down on the amount of time you spent on work or other activities during the past 4 weeks as a result of your physical health?
4b	RP 14	Blue	Did you accomplish less than you would like during the past 4 weeks as a result of your physical health?
4c	RP 15	Blue	Were you limited in the kind of work or other activities during the past 4 weeks as a result of your physical health?
4d	RP 16	Blue	Did you have difficulty performing the work or other activities during the past 4 weeks as a result of your physical health (for example, it took extra effort)?
5a	RE 17	Green	Did you cut down the amount of time you spent on work or other activities during the past 4 weeks as a result of any emotional problems?
5b	RE 18	Green	Did you accomplish less than you would like during the past 4 weeks as a result of any emotional problems?
5c	RE 19	Green	Did you not do work or other activities as carefully as usual during the past 4 weeks as a result of any emotional problems?
6	SF 20	Grey	During the past 4 weeks, to what extent has you physical health or emotional problems interfered with your normal social activities with family, friends, neighbors or groups?
7	BP 21	Purple	How much bodily pain have you had during the past 4 weeks?
8	BP 22	Purple	During the past 4 weeks, how much did pain interfere with your normal work (including both work outside the home and housework)?
9a	VT 23	Pink	How much of the time during the past 4 weeks did you feel full of pep?
9b	MH 24	Orange	How much of the time during the past 4 weeks have you been a very nervous person?
9c	MH 25	Orange	How much of the time during the past 4 weeks have you felt so down in the dumps that nothing would cheer you up?
9d	MH 26	Orange	How much of the time during the past 4 weeks have you felt calm and peaceful?
9e	VT 27	Pink	How much of the time during the past 4 weeks did you have a lot of energy?
9f	MH 28	Orange	How much of the time during the past 4 weeks have you felt downhearted and blue?
9g	VT 29	Pink	How much of the time during the past 4 weeks did you feel worn out?
9h	MH 30	Orange	How much of the time during the past 4 weeks have you been a happy person?
9i	VT 31	Pink	How much of the time during the past 4 weeks did you feel tired?
10	SF 32	Grey	During the past 4 weeks, how much of the time has your physical health or emotional problems interfered with your social activities (like visiting friends, relatives etc.)?
11a	GH 33	Red	How true or false is the statement “I seem to get sick a little easier than other people”?
11b	GH 34	Red	How true or false is the statement “I am as healthy as anybody I know”?
11c	GH 35	Red	How true or false is the statement “I expect my health to get worse”?
11d	GH 36	Red	How true or false is the statement “my health is excellent”?
Diagnostic status	DS	Brown	Dummy variable to distinguish between participants diagnosed with cancer (0) or not (1)

The domain colors correspond with node colors in Fig. 2a–c

As seen in Fig. 2a–c the global structure of each network reflects the domains set up by Ware et al. [41]; items that belong to the same domain are closely connected and cluster into predetermined domains.

These results comply with the idea that the covariance between items may result largely from direct interactions between observables, rather than from the common influence of a latent HRQoL variable. For instance, items 5a and 5b are strongly connected, which likely reflects a potential causal relation, because being able to spend less time on work will typically lead one to accomplish less. Another example is the strong connection between items 7 and 8, which is visible within all networks. However, there are also connections that are more likely to reflect bidirectional influences. An example is the relation between items 9c and 9f, where feeling down in the dumps and feeling downhearted and blue influence each other. Finally, some strong connections arise because items formulate necessary conditions for other items (exemplifying potential deterministic causal relations). For instance, there exists a strong correlation between items 3g and 3h. This correlation plausibly arises because walking 1 km requires the ability to walk a few 100 m, such that the latter is a necessary condition for the former.

**Fig. 3:**
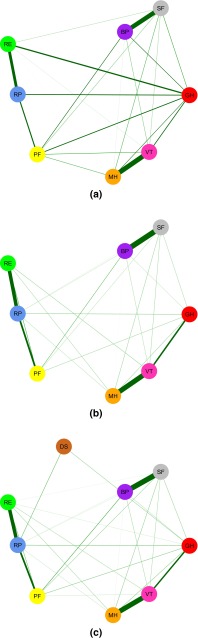
Network of Health-Related Quality of Life (HRQoL) as measured by the domains of the 36-item Short Form Health Survey in a cancer patient sample (**a**), a national sample (**b**) a pooled sample of the former two (**c**). The size of the absolute partial correlation between two nodes is represented using the *color* and thickness of an edge [37]. Node colors correspond to the eight domains: *RED* general health (GH), *YELLOW* physical functioning (PF), *ORANGE* mental health (MF), *BLUE* role limitations–physical (RP), *GREEN* role limitations–emotional (RE), *PURPLE* bodily pain (BP), *GREY* social functioning (SF), *PINK* vitality (VT), *BROWN* diagnostic status (DS)

Figure 3a–c shows networks of the eight domains present in the SF-36 for the cancer patient sample, the national sample and the combined sample. It can be seen that domains whose items are near each other in Fig. 2 are strongly connected. For example, the domains mental health (MH) and vitality (VT) have a strong connection in all networks. Interestingly, there exists a strong connection between bodily pain (BP) and social functioning (SF), while this is not visible in Fig. 2, where item networks are displayed.

### Network comparison

We compared the item network structures from both the cancer patient sample and the national sample. We found that these two network structures are dissimilar (*p* < .001) when comparing weighted network structures, but we did not find dissimilarity when comparing the unweighted network structures (*p* = .056). Although care must be taken in interpreting null results in hypothesis testing, this suggests that the basic structure of the SF-36 in the cancer patient sample resembles the structure found in the national sample. However, it should be noted that this does not rule out the existence of local differences in the network structure, as statistical power to detect local differences is limited.

The same analysis was performed for the domain networks. We could not reject the null hypothesis that the network structure is invariant over subpopulations, when comparing the unweighted network structures (*p* = .16) as well as when we compared the unweighted network structures (*p* = .18). Results indicate that the domain network structure generalizes to different subpopulations quite well.

### Centrality analysis

**Fig. 4:**
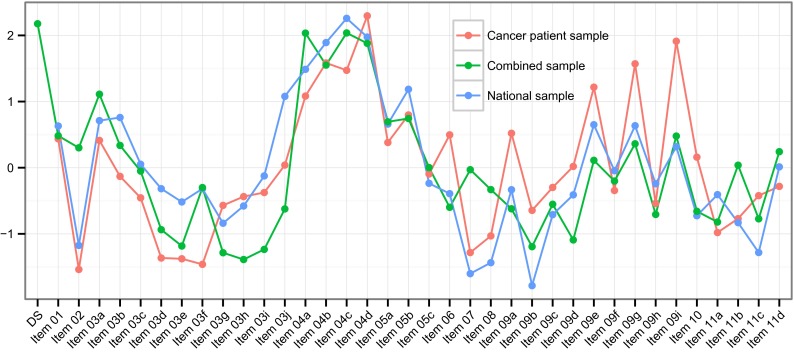
Visual representation of the predictive quality of individual Health-Related Quality of Life characteristics of the 36-item Short Form Health Survey in the network structures using the closeness centrality measure. *DS* diagnostic status

Figure 4 and Table 4 display the closeness centrality measures for all item networks. When inspecting closeness centrality, we find that the three networks mostly agree on which items are most central. As seen in Table 4, items 4b, 4c, 4d, 9g and 9i were most central for the cancer patient sample, items 4a, 4b, 4c, 4d and 5b were most central for the national sample and items 4a, 4b, 4c, 4d and the diagnostic status variable were most central for the combined sample. Items 4b, 4c and 4d are the items that are among the most central items in all networks. This suggests that in all datasets, the ability to keep participating in work or other activities despite one’s physical health has the largest influence on other characteristics in all networks. The networks align less with respect to the least central items. Items 2, 3d, 3e, 3f, and 7 were least central for the cancer patient sample, items 2, 7, 8, 9b and 11c were least central for the national sample and items 3e, 3g, 3h, 3i and 9b were least central for the combined sample. There were no items that were among the least central items in all networks, but items 2 and 7 were among the least central items in both the cancer patient sample and the national sample. Remarkably, this suggests that one’s perception of one’s health compared to 1 year ago and the amount of bodily pain during the past 4 weeks hardly influence other characteristics in the cancer patient sample network and the national sample network.

**Table 4:** Closeness centrality measure for every network to express the predictive quality of individual Health-Related Quality of Life characteristics in the network structure per sample in the 36-item Short Form Health Survey

Item	CaS	NaS	CoS
1	0.436	0.631	0.483
2	−1.538^b^	−1.175^b^	0.301
3a	0.412	0.712	1.111
3b	−0.131	0.759	0.337
3c	−0.455	0.052	−0.051
3d	−1.365^b^	−0.317	−0.937
3e	−1.377^b^	−0.518	−1.184^b^
3f	−1.461^b^	−0.318	−0.300
3g	−0.569	−0.839	−1.285^b^
3h	−0.437	−0.579	−1.388^b^
3i	−0.375	−0.124	−1.235^b^
3j	0.040	1.078	−0.623
4a	1.083	1.488^a^	2.035^a^
4b	1.584^a^	1.892^a^	1.551^a^
4c	1.473^a^	2.258^a^	2.038^a^
4d	2.296^a^	1.975^a^	1.880 ^a^
5a	0.381	0.659	0.694
5b	0.798	1.186^a^	0.744
5c	−0.095	−0.236	0.002
6	0.497	−0.392	−0.599
7	−1.283^b^	−1.602^b^	−0.03
8	−1.031	−1.435^b^	−0.331
9a	0.520	−0.333	−0.621
9b	−0.645	−1.783^b^	−1.194^b^
9c	−0.298	−0.71	−0.553
9d	0.019	−0.411	−1.092
9e	1.217	0.650	0.111
9f	−0.345	−0.045	−0.202
9g	1.571^a^	0.635	0.362
9h	−0.540	−0.243	−0.706
9i	1.912^a^	0.317	0.478
10	0.160	−0.726	−0.659
11a	−0.980	−0.407	−0.821
11b	−0.770	−0.831	0.038
11c	−0.422	−1.282^b^	−0.773
11d	−0.283	0.013	0.242
Diagnostic status	–	–	2.177^a^

All values are standardized and comparable

*CaS* cancer patient sample, *NaS* national sample, *CoS* combined sample

^a^Top five highest closeness

^b^Top five lowest closeness

**Fig. 5:**
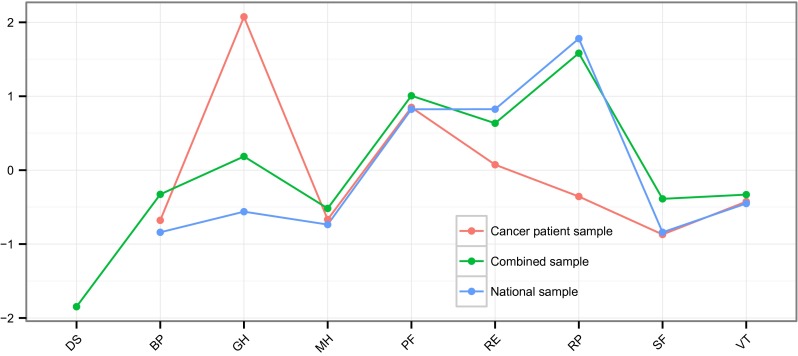
Visual representation of the predictive quality of domains of the 36-item Short Form Health Survey in the network structures using the closeness centrality measures. *BP* bodily pain, *GH* general health, *MH* mental health, *PF* physical functioning, *RE* role limitations–emotional, *RP* role limitations–physical, *SF* social functioning, *VT* vitality, *DS* diagnostic status

Figure 5 and Table 5 display the closeness centrality measures for all domain networks. When inspecting closeness centrality, we find that the three networks disagree on which items are most central. As found in Table 5, the domains general health (GH) and BP are most central in the cancer patient sample, the domains role limitations–physical (RP) and role limitations–emotional (RE) in the national sample, and the domains RP and PF in the combined sample. The cancer patient sample and the national sample do not share any domain that is among the most central domains in the networks. The domain PF is among the most central domains in both the cancer patient sample and the combined sample, and the domain RP is among the most central domains in the national sample and the combined sample. This suggests that physical health and possible limitations as a result of one’s physical health have the largest influence on other domains in the networks.

**Table 5:** Closeness centrality measure for every network to express the predictive quality of domains of the 36-item Short Form Health Survey in the network structure per sample

Domain	CaS	NaS	CoS
BP	−0.678^b^	−0.840^b^	−0.327
GH	2.074^a^	−0.562	0.186
MH	−0.667	−0.736	−0.517^b^
PF	0.849^a^	0.824	1.007^a^
RE	0.074	0.825^a^	0.635
RP	−0.356	1.780^a^	1.583^a^
SF	−0.870^b^	−0.840^b^	−0.388
VT	−0.426	−0.451	−0.331
Diagnostic Status	–	–	−1.847^b^

All values are standardized and comparable

*CaS* cancer patient sample, *NaS* national sample, *CoS* combined sample

^a^Top two highest closeness

^b^Top two lowest closeness

The same non-alignment is found in the least central domains. Domains SF and BP are considered the least central domains in the cancer patient sample and the national sample, and the domain MH and the diagnostic status variable (DS) were the least central domains in the combined sample. It can be seen that the cancer patient sample and the national sample regard the same domains as least central. This suggests that the interaction between social functioning and bodily pain on the one hand, and the rest of the domains on the other hand, is less strong compared to other interactions in the network. This may mean that they have the least influence on other domains in the network, that they are least sensitive to changes in other domains or that the variance in these domains is largely determined by factors outside of the network structure.

## Discussion

The present study demonstrated a new approach for modeling HRQoL, in which HRQoL emerges from a network of mutually interacting characteristics. We provided the first estimated network structure for HRQoL, by determining the GGM for the SF-36. In this network, every pairwise interaction is evaluated while controlling for all other variables in the network, after which the network is regularized by a lasso penalization. Edges that survive the resulting process of culling thus are likely to have a causal background. Importantly, the present analysis does not determine what the nature or direction of that causal background is. For example, some relationships between items may reflect potential causal effects, while others may be potential bidirectional relationships or potential conditional relationships that exemplify nearly deterministic relations. In addition, some items may hang together because they depend on one or a set of unmodeled latent variables.

In case unmodeled latent variables affect multiple items simultaneously, this will generate a fully connected sub network or *clique* in the network [42]. However, in our view it is extremely unlikely that all connections result from a common latent structure (as the RMM assumes). However, it would be worthwhile to develop analytical techniques that can combine latent variables analysis with network modeling. For now, the NM models relevant associations in a manner that is both statistically efficient and may also be more justifiable than the RMM, as it does not force a particular causal model to the data. Given our limited understanding of constructs like HRQoL, further explanation of network analysis as a tool in HRQoL research is therefore warranted.

As research advances, and the field improves its understanding of the causal relations that underlie the network structure of HRQoL, NMs can identify the most important nodes in the structure, as we have shown. By crossing the effect of variables on other variables in the network with the cost and availability of interventions directed at these variables, NMs may inform treatment decisions. Centrality analyses showed that the ability to perform work or other activities and accomplish things despite one’s physical health was most central in all network structures. This is indeed a plausible conclusion, given the importance of maintaining a daily routine in people’s lives. Furthermore, results showed that physical health and possible limitations as a result of one’s physical health are central domains in the domain network. In terms of treatment, these findings suggest that it is important to have access to direct resources that allow people to keep their daily routine and perform work or other activities as usual, as doing so may stop vicious circles from within the network structure. Thus, in the future, NMs may bridge the gap between research and treatment practice by providing specific guidance on treatment interventions.

Moreover, we found similar, unweighted, item network structures for both samples, next to the domain networks that were equivalent in for both weighted and unweighted networks. Even though the domains’ network structure is similar, network structures on item level may differ over distinct groups (e.g., age, depression). Investigating group differences may offer important inroads to understanding differential treatment effects or group differences in vulnerability. Future research may focus on relating the network structure extracted in the current study with networks that characterize other subpopulations.

In conclusion, this study supports the further explanation of NMs as a tool in HRQoL research and highlights the need for more research into comparison and confirmatory methods for network modeling, as this would help to compare networks across subpopulations and to generalize network structures to larger populations. In addition, NMs may be coupled to the analysis of treatment interventions. Thus, we propose that investigating the network structure of HRQoL will allow research to advance by taking advantage of the many possibilities that NMs have to offer.

## References

[1] Edwards JR, Bagozzi RP (2000). On the nature and direction of relationships between constructs and measures. Psychological Methods.

[2] Kieffer JM, Verrips E, Hoogstraten J (2009). Model specification in oral health-related quality of life research. European Journal of Oral Sciences.

[3] De Vet HCW, Adèr HJ, Terwee CB, Pouwer F (2005). Are factor analytical techniques used appropriately in the validation of health status questionnaires? A systematic review on the quality of factor analysis of the SF-36. Quality of Life Research.

[4] Ware JE, Sherbourne CD (1992). The MOS 36-item short-form health survey (SF-36): I. Conceptual framework and item selection. Medical Care.

[5] Wright S, Blalock HM (1971). Path coefficients and path regressions: alternative or complementary concepts?. Causal models in the social sciences.

[6] Borsboom D, Markus K (2013). Truth and evidence in validity theory. Journal of Educational Measurement.

[7] Howell RD, Breivik E, Wilcox JB (2007). Reconsidering formative measurement. Psychological Methods.

[8] Wolinsky FD, Stump TE (1996). A measurement model of the Medical Outcomes Study 36-Item Short-Form Health Survey in a clinical sample of disadvantaged, older, black, and white men and women. Medical Care.

[9] Wu C, Lee K, Yao G (2007). Examining the hierarchical factor structure of the SF-36 Taiwan version by exploratory and confirmatory factor analysis. Journal of Evaluation in Clinical Practice.

[10] Bollen KA, Lennox R (1991). Conventional wisdom on measurement: A structural equation perspective. Psychological Bulletin.

[11] Borsboom D (2005). Measuring the mind: Conceptual issues in contemporary psychometrics.

[12] Fayers PM, Hand DJ (1997). Factor analysis, causal indicators and quality of life. Quality of Life Research.

[13] Fayers PM, Hand DJ, Bjordal K, Groenvold M (1997). Causal indicators in quality of life research. Quality of Life Research.

[14] Eaton NR (2015). Latent variable and network models of comorbidity: toward an empirically derived nosology. Social Psychiatry and Psychiatric Epidemiology.

[15] Koller D, Friedman N (2009). Probabilistic graphical models: principles and techniques.

[16] Van der Maas HLJ, Dolan CV, Grasman R, Wicherts JM, Huizenga HM, Raijmakers MEJ (2006). A dynamical model of general intelligence: The positive manifold of intelligence by mutualism. Psychological Review.

[17] Cramer AOJ, Waldorp LJ, van der Maas HLJ, Borsboom D (2010). Comorbidity: A network perspective. Behavioral and Brain Sciences.

[18] Cramer AOJ, Sluis S, Noordhof A, Wichers M, Geschwind N, Aggen SH (2012). Dimensions of normal personality as networks in search of equilibrium: You can’t like parties if you don’t like people. European Journal of Personality.

[19] Whittaker J (1990). Graphical models in applied multivariate statistics.

[20] Aaronson NK, Muller M, Cohen PDA, Essink-Bot M-L, Fekkes M, Sanderman R (1998). Translation, validation, and norming of the Dutch language version of the SF-36 Health Survey in community and chronic disease populations. Journal of Clinical Epidemiology.

[21] Ware JE, Gandek B (1998). Overview of the SF-36 health survey and the international quality of life assessment (IQOLA) project. Journal of Clinical Epidemiology.

[22] Ware JE (2000). SF-36 health survey update. Spine.

[23] Jenkinson C, Wright L, Coulter A (1994). Criterion validity and reliability of the SF-36 in a population sample. Quality of Life Research.

[24] Ware JE, Kosinski M, Gandek B, Aaronson NK, Apolone G, Bech P (1998). The factor structure of the SF-36 Health Survey in 10 countries: results from the IQOLA Project. Journal of Clinical Epidemiology.

[25] Brazier J, Harper R, Jones N, O’cathain A, Thomas K, Usherwood T, Westlake L (1992). Validating the SF-36 health survey questionnaire: new outcome measure for primary care. British Medical Journal.

[26] McHorney CA, Ware JE, Raczek AE (1993). The MOS 36-Item Short-Form Health Survey (SF-36): II. Psychometric and clinical tests of validity in measuring physical and mental health constructs. Medical Care.

[27] Ware JE, Kosinski M, Keller SD (1994). SF-36 physical and mental health summary scales: A user’s manual.

[28] Borsboom D (2008). Psychometric perspectives on diagnostic systems. Journal of Clinical Psychology.

[29] Newman M (2009). Networks: An introduction.

[30] Lauritzen SL, Wermuth N (1989). Graphical models for associations between variables, some of which are qualitative and some quantitative. The Annals of Statistics.

[31] Friedman JH, Hastie T, Tibshirani R (2008). Sparse inverse covariance estimation with the graphical lasso. Biostatistics.

[32] Tibshirani R (1996). Regression shrinkage and selection via the lasso. Journal of the Royal Statistical Society. Series B (Methodological).

[33] Foygel R, Drton M, Lafferty JD, Williams CKI, Shawe-Taylor J, Zemel RS, Culotta A (2010). Extended Bayesian information criteria for Gaussian graphical models. Advances in neural information processing systems 23.

[34] Costantini, G., Epskamp, S., Borsboom, D., Perugini, M., Mõttus, R., Waldorp, L. J., & Cramer, A. O. J. (2015). State of the aRt personality research: A tutorial on network analysis of personality data in R. *Journal of Research in Personality*, *54*, 13–29.

[35] Borkulo van, C. D., Waldorp, L. J., Boschloo, L., Schoevers, R. A., & Borsboom, D. (2015). *Statistical comparison of two networks with respect to global strength*. Retrieved from https://github.com/cvborkulo/NetworkComparisonTest.

[36] Barrat A, Barthelemy M, Romualdo P-S, Vespignani A (2004). The architecture of complex weighted networks. Proceedings of the National Academy of Sciences of the United States of America.

[37] Opsahl T, Agneessens F, Skvoretz J (2010). Node centrality in weighted networks: Generalizing degree and shortest paths. Social Networks.

[38] Zhao, T., Liu, H., Roeder, K., Lafferty, J., & Wasserman, L. (2014). *Huge: High-dimensional undirected graph estimation*. Retrieved from http://cran.r-project.org/package=huge. PMC4729207 26834510

[39] Epskamp, S., Cramer, A. O. J., Waldorp, L. J., Schmittmann, V. D., & Borsboom, D. (2012). qgraph: Network visualizations of relationships in psychometric data. *Journal of Statistical Software*, *48*(4), 1–18. Retrieved from http://www.jstatsoft.org/v48/i04/.

[40] Fruchterman TMJ, Reingold EM (1991). Graph drawing by force-directed placement. Software: Practice and Experience.

[41] Ware JE, Gandek B, Kosinski M, Aaronson NK, Apolone G, Brazier JE (1998). The equivalence of SF-36 summary health scores estimated using standard and country-specific algorithms in 10 countries: results from the IQOLA project. Journal of Clinical Epidemiology.

[42] Marsman M, Maris G, Bechger T, Glas C (2015). Bayesian inference for low-rank Ising networks. Scientific Reports.

